# Stepwise Reversion of Multiply Mutated Recombinant Antitrypsin Reveals a Selective Inhibitor of Coagulation Factor XIa as Active as the M358R Variant

**DOI:** 10.3389/fcvm.2021.647405

**Published:** 2021-03-19

**Authors:** Mostafa Hamada, Varsha Bhakta, Sara N. Andres, William P. Sheffield

**Affiliations:** ^1^Department of Pathology and Molecular Medicine, McMaster University, Hamilton, ON, Canada; ^2^Centre for Innovation, Canadian Blood Services, Hamilton, ON, Canada; ^3^Department of Biochemistry and Biomedical Sciences, McMaster University, Hamilton, ON, Canada

**Keywords:** serpins, inhibitors, blood coagulation factors, antitrypsin, molecular modeling, recombinant proteins

## Abstract

Alpha-1 antitrypsin (AAT, also known as alpha-1 proteinase inhibitor or SERPINA1) is the most abundant member of the serpin superfamily found in human plasma. The naturally occurring variant AAT M358R, altered at the P1 position of the critical reactive center loop (RCL), is re-directed away from inhibition of AAT's chief natural target, neutrophil elastase, and toward accelerated inhibition of thrombin (FIIa), kallikrein (Kal), and other proteases such as factor XIa (FXIa). FXIa is an emerging target for the development of antithrombotic agents, since patients with FXI deficiency are protected from thromboembolic disease and do not exhibit a strong bleeding tendency. Previously, we used phage display, bacterial lysate screening, and combinatorial mutagenesis to identify AAT-RC, an engineered AAT M358R with additional changes between RCL positions P7-P3', **C**LE**VE**P**R-**S**TE** [with changes bolded and the P1-P1' (R358-S359) reactive center shown as R-S]. AAT-RC was 279- and 16-fold more selective for FXIa/IIa or FXIa/Kal than AAT M358R; the increased selectivity came at a cost of a 2.3-fold decrease in the rate of FXIa inhibition and a 3.3-fold increase in the stoichiometry of inhibition (SI). Here, we asked which alterations in AAT-RC were most important for the observed increases in selectivity for FXIa inhibition. We back-mutated AAT-RC to AAT-RC-1 (P7-P3' FLE**VE**P**R**S**TE**), AAT-RC-2 (P7-P3' FLEA**E**P**R**S**TE**), and AAT RC-3 (P7-P3' FLEAIP**R**-S**TE**). Proteins were expressed as cleavable, hexahistidine-tagged glutathione sulfotransferase fusion proteins in *E. coli* and purified by proteolytic elution from glutathione agarose, with polishing on nickel chelate agarose. Selectivity for FXIa over Kal of AAT-RC-1, −2, and −3 was 14, 21, and 2.3, respectively. AAT-RC-2 inhibited FXIa 31% more rapidly than AAT M358R, with the same SI, and enhanced selectivity for FXIa over Kal, FXa, FXIIa, activated protein C, and FIIa of 25-, 130-, 420-, 440-, and 470-fold, respectively. Structural modeling of the AAT-RC-2/FXIa encounter complex suggested that both E (Glu) substitutions at P3 and P3' may promote FXIa binding via hydrogen bonding to K192 in FXIa. AAT-RC-2 is the most selective and active AAT variant reported to date for FXIa inhibition and will be tested in animal models of thrombosis and bleeding.

## Introduction

Circulating at a concentration of ~20 μM, α_1_-antitrypsin (AAT, also known as alpha-1 proteinase inhibitor or SERPIN A1) is the most abundant member of the serpin superfamily found in human plasma (1–3). Although the principal physiological target of AAT is neutrophil elastase (3, 4), inhibitory complexes of AAT and other proteases such as activated coagulation factor XI (FXIa) can be found in human plasma (5, 6). While the rate of FXIa inhibition by native AAT is relatively slow (7), a naturally occurring M358R (AAT Pittsburgh) substitution in its critical reactive center loop (RCL) increases its rate of inhibition > 1,000-fold for FXIa and other clotting-related factors, while similarly reducing its inhibition of neutrophil elastase (8–10). FXI has emerged as an attractive target for the development of antithrombotic agents with improved therapeutic profiles (11), because reductions in FXI or FXIa levels correlate with lower thrombotic risk without provoking hemorrhage in mice (12–14) and humans (15–20). We hypothesized that mutating additional residues in the RCL of AAT M358R would yield a specific FXIa inhibitor. Although multiple anti-FXI and anti-FXIa agents of either a small molecule or macromolecular nature are in pre-clinical or clinical development, none has as yet reached licensure (21), and a serpin-based FXIa inhibitor could have advantages with respect to low toxicity and relatively rapid off-set of action.

Mechanistically, serpins present their RCL residues to attacking proteases, forming an encounter complex that proceeds through a tetrahedral intermediate stage to cleavage of the reactive center peptide bond (2, 22, 23). Bond scission releases free energy and powers a large conformational change in which the protease, still connected to the serpin RCL via an acyl ester bond between the RCL and its active site, is translocated to the opposite pole of the serpin, as the cleaved RCL inserts into the central β-sheet of the serpin as a new strand (2, 22, 23). The trapped protease is distorted in its active site and its ability to complete catalysis is severely impaired. Structural biological support for this model has been provided by crystal structures of multiple serpins, including the non-covalent complex of AAT M358R and active site-mutated S195A trypsin (24) and the covalent complexes of AAT with trypsin (25) or elastase (26). In terms of reaction outcomes, serpins exhibit a branched reaction pathway leading either to covalent complex formation or to the release of active protease and inactive serpin cleaved within its RCL (27, 28). While antithrombin is the principal serpin regulating coagulation, other serpins such as C1 inhibitor, protein C inhibitor, and protease nexin 1 also play some roles in this pathway (29).

While many previous investigations [reviewed in Scott and Sheffield (30)] have attempted to change the specificity of AAT to react with different target proteases, via directed mutagenesis or loop exchanges, few have addressed re-orienting AAT to inhibit FXIa specifically. Previously, we divided the AAT RCL into three sectors, screening hypervariable phage display or bacterial lysate libraries for motifs that provided more specific inhibition of FXIa than other proteases (31). We found that the combination of the second and third motifs, in AAT variant AAT-RC, containing five substitutions additional to M358R in its RCL (F352C/A355V/I356E/I360T/P361E), increased FXIa selectivity over thrombin, FXIIa, FXa, activated protein C, and kallikrein by 279-, 143-, 63-, 58-, and 36-fold, respectively, vs. AAT M358R. AAT-RC inhibited FXIa 2.3-fold less rapidly than AAT M358R and exhibited a 3.3-fold increase in reaction stoichiometry (31). In the current study, we back-mutated AAT-RC, one residue at a time, toward AAT M358R, to ascertain if less mutated variants retained or exceeded AAT-RC's activity against and selectivity for FXIa. We report that a variant with three substitutions additional to M358R, AAT-RC-2 (I356E/M358R/I360T/P361E), retained not only the full activity and reaction stoichiometry of AAT M358R but also the FXIa selectivity of AAT-RC.

## Methods

### Reagents

The following coagulation and coagulation-related proteases were purchased from Enzyme Research Labs (USA): kallikrein and thrombin [also called factor (F) IIa, FXa, FXIa, and FXIIa]. The following chromogenic substrates were purchased from Diapharma (USA): for thrombin, S-2238; for FXa, S-2765; for FXIa and APC, S-2366; and for kallikrein and FXIIa, S-2302. Custom synthetic double-stranded DNA fragments (gBlocks) were bought from Integrated DNA Technologies (Canada). Restriction endonucleases and glutathione agarose were purchased from Thermo Fisher Scientific (Canada). Nickel chelate affinity resin Ni-NTA agarose was bought from Qiagen (Canada). PreScission Protease [a glutathione sulfotransferase (GST)–human rhinovirus (HRV) 3C protease fusion protein] was purchased from GE Healthcare (Canada). Normal human pooled plasma (NHPP) was produced in-house. FXI-deficient plasma was purchased from Affinity Biologicals (Canada). STA PTTA reagent, STA Neoplastine CI Plus reagent, and Owren-Koller buffer were bought from Diagnostica Stago (Canada).

### DNA Manipulations

Synthetic DNA fragments of 941 bp in length were designed to encode each of the AAT variants listed in Table 1: AAT-RC-1, AAT-RC-2, and AAT-RC-3. Following restriction with *Kpn2I* and *EcoRI*, the resulting 925-bp fragments were each separately ligated to the 5256-bp *Kpn2I*/*EcoRI* double digestion product of pGEX-AAT M358R (31) to create pGEX-AAT-RC-1, −2, or −3, respectively. Ligation reactions were used to transform *E. coli* DH5α to ampicillin resistance, and subclones were screened by standard methods as described (32, 33). Each plasmid construct was validated by DNA sequencing using the Sanger method by Mobix Lab, a central facility of the Faculty of Health Sciences, McMaster University. These plasmids encoded a 630-amino acid glutathione sulfotransferase (GST)–AAT fusion protein linking GST C-terminal K218 to Glu1 of AAT via peptide SDLEVLFQ-GPMGH_6_, which specified a PreScission Protease recognition site and a hexahistidine tag. Validated plasmids were transferred to *E. coli* BL21 for protein expression as previously described (31).

**Table 1:** RCL sequences of AAT M358R and variants examined or considered in this study.

**Protein name**	**RCL sequence (P13-P3')**
AAT-RC^a^	EAAGAM**C**LE**VE**PRS**TE**
AAT-RC-1	EAAGAMFLE**VE**PRS**TE**
AAT-RC-2	EAAGAMFLEA**E**PRS**TE**
AAT-RD^a^	EAAGAMFLEAIPRS**TE**
AAT-RC-3	EAAGAMFLEAIPRSI**E**
AAT M358R	EAAGAMFLEAIPRSIP

*Primary amino acid sequences of various AAT M358R derivatives between P7 (F352) and P3' (P361) are shown. Regular font indicates AAT M358R sequence; bolded and underlined font indicates mutated residues*.

a *Previously reported (31)*.

### Protein Expression and Purification

*E. coli* BL21 transformed with pGEX-AAT M358R, -RC, -RC-1, −2, or −3 was grown at 37°C to mid-log phase while shaking at 225 rpm, and GST–AAT fusion protein expression was induced with 0.1 mM isopropyl β-D-1-thiogalactopyranoside (IPTG). AAT proteins were purified from lysates liberated from cell pellets by sonication and purified via glutathione agarose chromatography with proteolytic elution of AAT variants with PreScission Protease and polishing via nickel chelate affinity chromatography as described (31).

### Kinetic Analysis

Pseudo-first-order conditions, comprising at least a 10:1 molar ratio of AAT variant to protease, were employed to determine the second-order rate constant of inhibition (*k*_2_) of different proteases (5–75 nM) by AAT variants at 37°C, employing a previously described discontinuous method (31–33). Briefly, this approach involved dividing the observed rate constant *k*_obs_, which was the slope of the plot of the natural logarithm of the ratio of initial protease activity to residual protease activity vs. time, by the initial AAT variant molar concentration. Similarly, the stoichiometry of inhibition (SI) for FXIa was determined as previously described (31–33). Briefly, this approach involved incubating varying ratios of AAT variants to FXIa at ambient temperature for 2 h, determining the residual FXIa activity at 37°C, plotting it vs. the ratio of AAT/FXIa, and regressing the resulting line of best fit to zero residual FXIa activity to yield the SI.

### Gel-Based Analysis

The electrophoretic profile of the reaction of AAT variants with thrombin or FXIa was visualized on 10% sodium dodecyl sulfate (SDS)-polyacrylamide gels, except where indicated, under reducing conditions. In reactions with thrombin or FXIa with a total volume of 15 μl, AAT variants (1.0 μM final concentration) were reacted with 100 nM protease in 20 mM sodium phosphate, pH 7.4, 0.1% polyethylene glycol 8000, 100 mM NaCl, and 0.1 mM EDTA (PPNE buffer) for 0 (no protease control), 1 or 5 m at 37°C. Reactions were stopped by the addition of 1/3 reaction volume of concentrated SDS sample buffer as described (32), and samples containing the entire reaction volume were electrophoresed. In some reactions with FXIa, the reaction time was fixed at 5 min and molar ratios of AAT/FXIa were varied (10:1, 2:1, 1:1, and 1:2) while keeping the AAT variant concentration fixed at 1.0 μM. Gels were stained with Coomassie Brilliant Blue and destained as described (31). Gels were scanned using a model XR Gel Doc system manufactured by Bio-Rad Laboratories (Canada). Captured images were labeled and saved in a Tagged Image File (TIF) format using PhotoShop CS6 version 13 software from Adobe Systems Incorporated (USA). Image brightness was not manipulated unless so stated in the figure legend.

### Diluted Prothrombin Time Assay

The prothrombin time (PT) assay was modified by dilution of test reagents essentially as done by de Maat and co-workers (34). PT reagent STA Neoplastine CI Plus (Diagnostica Stago) was diluted 1:500 with 16.6 mM CaCl2, and 0.1 ml of the diluted reagent was combined with 0.05 ml of normal human pooled plasma (NHPP) supplemented with purified AAT variants. Both test solutions were pre-warmed to 37°C prior to combination in a STA-IV clotting analyzer (Diagnostica Stago) and determination of clotting time.

### Diluted APTT Assays

Standard APTT assays were modified in by dilution of the APTT reagent. The APTT reagent (STA PTTA) was diluted 1:15 with Owren-Koller buffer. NHPP supplemented with AAT variant protein (0.05 ml) was then combined with 0.05 ml of diluted reagent and pre-warmed to 37°C. Clotting was then initiated by the addition of 0.05 ml of 25 mM CaCl_2_, and clotting time was determined on the analyzer specified above (31).

### Protein Modeling

The encounter or Michaelis complexes between AAT M358R, AAT-RC, and AAT-RC-2 were separately modeled using PyMOL Molecular Graphics System 2.3.4 (https://pymol.org) and ClusPro 2.0 (https://cluspro.bu.edu) (35–37). Protein Data Bank (PDB) file 1OPH of the non-covalent complex between AAT M358R and active site-mutated S195A trypsin (24) was first manipulated to select chain A (AAT M358R) and then to introduce the five mutations of AAT-RC or the three mutations of AAT-RC-2, using the backbone-dependent rotamer feature in PyMOL to minimize steric clashes. In ClusPro 2.0, AAT M358R from 1OPH or mutated PDB files based on 1OPH were designated the receptor, and the catalytic domain of FXIa (FXIac) from chain A of PDB 1ZJD was designated the ligand (38). Residues between AAT E346 and P361 or mutated equivalents and FXIac residues 57, 98, 102, 143, 151, 189, 192, 193, and 195 were selected for attraction based on crystal structural (38) or mutational information (39) identifying them as forming ionic or hydrogen bonds with the co-crystalized Kunitz protease inhibitor domain of protease nexin 2 (KPI-PN2) in PDB 1ZJD; H57, D102, and S195 form the catalytic triad of the FXIac active site. The structures were then docked using ClusPro 2.0. Resulting models of the best conformational fit were chosen based on the balanced scoring scheme, using the central model from the largest sized cluster. Hydrogen bond lengths between P3 and P3' residues on AAT and K192 on FXIac (or distances if hydrogen bonding was not possible) were determined using PyMOL. Rendering of figures was performed using PyMOL.

### Statistical Analysis

Statistical analysis was facilitated using InStat version 3.06 (GraphPad Software, San Diego CA). Prism 4.03 (GraphPad Software) was employed to generate graphs. Multiple comparisons were performed using ANOVA with Tukey post-tests for data sets passing tests of normality and similarity of standard deviations and with non-parametric ANOVA with Dunn's post tests for those failing one or more tests. Comparisons returning a *p* < 0.05 were considered statistically significant.

## Results

### General Approach

Table 1 shows the amino acid sequence of the RCL of AAT variants analyzed in this study. The general mutational approach was to start with AAT-RC, containing five amino acid substitutions compared to AAT M358R between P7 (F352) and P3' (P361), and reverse the mutations, one at a time, back toward the AAT M358R sequence. In so doing, we generated novel proteins AAT-RC-1, −2, and −3 and compared them to previously described proteins AAT-RC and AAT-RD. The latter were selected from phage display or bacterial expression libraries via biopanning or lysate screening (31). All variants were expressed in *E. coli*, purified to homogeneity, and reacted with different proteases to assess inhibition.

It should be noted that we did not characterize each variant exhaustively. Rather, we employed a lean design in which we first compared the rates of FXIa inhibition of AAT-RC-1, −2, and −3 to AAT M358R and AAT-RC (31) and the selectivity for FXIa over kallikrein, as a selected representative of other proteases inhibited less rapidly by AAT-RC than AAT M358R (31). We then chose the most selective of these three variants for more detailed characterization.

### AAT RC-2 Selectively Inhibits FXIa Over Kallikrein More Effectively Than Other Variants

We first investigated the rate of inhibition of FXIa of the variants. As shown in Table 2, while AAT-RC-1, −2, and −3 each exhibited a greater mean *k*_2_ of FXIa inhibition than AAT-RC, the most rapid inhibitor of FXIa was AAT-RC-2. Reverting the F352C mutation to the native phenylalanine at P7 restored the decrease in the rate of FXIa inhibition observed between AAT M358R and AAT-RC; indeed, the mean *k*_2_ of AAT-RC-2 for FXIa was 32% greater than that of AAT M358R. With respect to inhibition of another serine protease inhibited by AAT M358R, kallikrein, the AAT-RC-1, −2, and −3 variants were 3–42-fold slower inhibitors of kallikrein than AAT M358R, but all inhibited kallikrein more rapidly than AAT-RC. The selectivity index, or the ratio of the rate constants for FXIa inhibition over those for kallikrein inhibition, was greatest for AAT-RC-2, at 142, and this variant was therefore selected for further study.

**Table 2:** Kinetic parameter AAT variants compared to AAT M358R.

**Protein**	***k*_**2**_ for FXIa (× 10^**5**^ M^**−1**^s^**−1**^)**	***k*_**2**_ for kallikrein (× 10^**5**^ M^**−1**^s^**−1**^)**	**Selectivity (*k*_**2**_ for FXIa/*k*_**2**_ for kallikrein)**	**Fold increase in kallikrein selectivity vs. AAT M358R**	**SI vs. FXIa**	**SI vs. kallikrein**
AAT M358R	1.4 ± 0.2^b^	0.21 ± 0.06^a^	6.67	–	2.7 ± 0.4^a^	3.4 ± 0.1^b^
AAT-RC	0.54 ± 0.09^b^	0.0050 ± 0.0002^a^	108	16.2	7 ± 1^d^	ND
AAT-RC-1	1.7 ± 0.2^b^	0.018 ± 0.002^b^	94	14.1	ND	ND
AAT-RC-2	1.85 ± 0.05^b^	0.013 ± 0.002^b^	142	21.3	2.7 ± 0.8^c^	64 ± 1^b^
AAT-RC-3	1.0 ± 0.1^b^	0.066 ± 0.006^b^	15.2	2.3	ND	ND

*The mean of 4^a^, 5^b^, 6^c^, or 8^d^ determinations ± one standard deviation is shown, for kinetic parameters for AAT variants, compared to AAT M358R*.

With respect to reaction stoichiometry, as shown in Table 2, bacterially expressed AAT M358R exhibited a mean SI of 2.7, indicating that 2.7 molecules of AAT M358R were required, on average, to inhibit a molecule of FXIa, under steady-state conditions. This value rose to 7 for AAT-RC or -RD, indicating a decreased efficiency of inhibition. In contrast, reducing the number of mutations to four in AAT-RC-2 restored the SI to the same mean value observed for AAT M358R, 2.7. Having demonstrated that AAT-RC-2 and AAT M358R had identical SI values, we asked if this was also true for kallikrein inhibition. In contrast to the results with FXIa, we found substantial elevation of SI for the reaction of kallikrein with AAT-RC-2 compared to FXIa (mean values of 64 vs. 3.3, Table 2).

**Figure 1:**
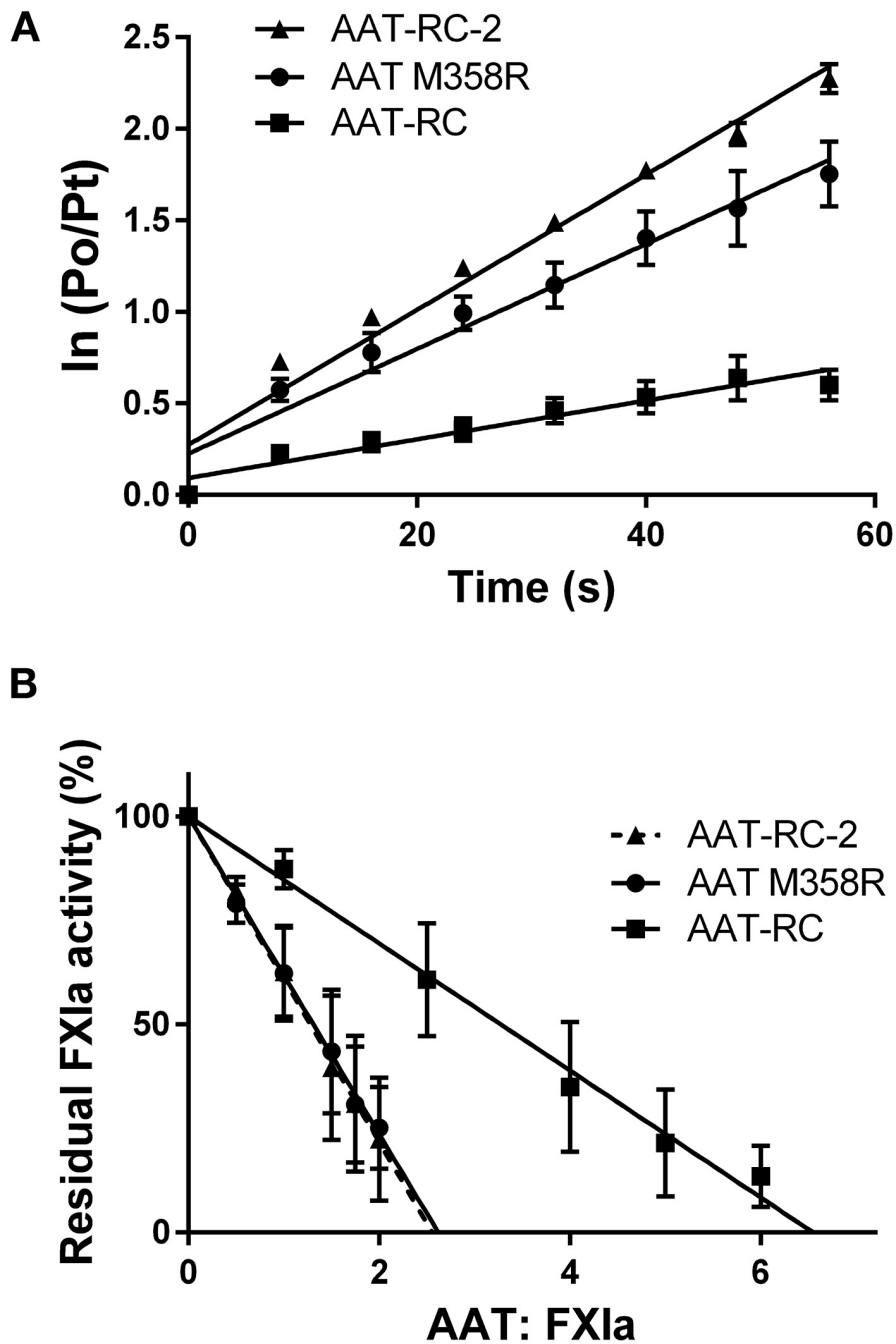
Kinetic parameters of AAT variants. **(A)** The natural logarithm of the ratio of the initial protease (FXIa) activity (*P*_0_) to the residual activity at time *t* (*P*_*t*_) vs. the time in seconds [time (s)]. Points are the mean ± SD of five determinations; lines are lines of best fit determined by linear regression. **(B)** The residual FXIa activity as a percentage of initial activity after incubation of AAT variants with differing molar ratios of FXIa for 2 h. Points are the mean of four (AAT M358R), six (AAT-RC-2), or eight (AAT-RC) separate experiments; lines are lines of best fit determined by linear regression. Note that the regression line for AAT-RC-2 is dashed to distinguish it from its superimposed neighbor, the line for AAT M358R.

Figure 1 shows plots used to determine *k*_2_ and SI values for the reaction of AAT M358R, AAT-RC, and AAT-RC-2 with FXIa, which are representative of all such kinetic determinations in this report and which correspond to the data shown in Tables 2, 3.

**Table 3:** Kinetic parameters for AAT-RC-2 and AAT M358R with various proteases.

**Protease**	***k*_**2**_ AAT M358R (× 10^**5**^ M^**−1**^s^**−1**^)**	***k*_**2**_ AAT-RC-2 (× 10^**5**^ M^**−1**^s^**−1**^)**	**Selectivity of AAT M358R (*k*_**2**_ for FXIa/*k*_**2**_ for protease)**	**Selectivity of AAT-RC-2 (*k*_**2**_ for FXIa/*k*_**2**_ for protease)**	**Fold difference in selectivity (AAT-RC-2/AAT M358R)**
FXIa	1.4 ± 0.2^c^	1.85 ± 0.05^e^	–	–	–
Kallikrein	0.24 ± 0.02^b^	0.013 ± 0.02^c^	5.8	142	25
Thrombin	1.8 ± 0.2^b^	0.005 ± 0.001^d^	0.78	370	470
APC	0.25 ± 0.03^a^	0.0007 ± 0.0003^b^	5.6	2,440	440
FXa	0.29 ± 0.03^d^	0.003 ± 0.001^d^	4.8	620	130
FXIIa	0.23 ± 0.04^d^	0.0005 ± 0.0002^d^	8.8	3,700	420

*The mean of 3^a^, 4^b^, 5^c^, 7^d^, or 11^e^ determinations ± one standard deviation is shown, for kinetic parameters for AAT-RC-2 and AAT M358R with various proteases*.

### AAT-RC-2 Selects FXIa Over Five Other Serine Proteases as Inhibitory Targets

The rates of inhibition of six serine proteases were next assessed for AAT M58R and AAT-RC-2. As shown in Table 3, mean *k*_2_ values for inhibition of kallikrein, thrombin, APC, FXa, and FXIIa were all substantially reduced for AAT-RC-2 compared to AAT M358R. Combined with the slight increase in the *k*_2_ of AAT-RC-2 for FXIa inhibition, these rate constants yielded selectivity factors of between 142 (kallikrein) and 3,700 (for FXIIa) for AAT-RC-2, which corresponded to increases in selectivity for the different proteases of AAT-RC-2 compared to AAT M358R ranging from 25-fold for kallikrein to 470-fold for thrombin.

### AAT-RC-2 Forms Denaturation-Resistant Inhibitory Complexes With FXIa but Not Thrombin

**Figure 2:**
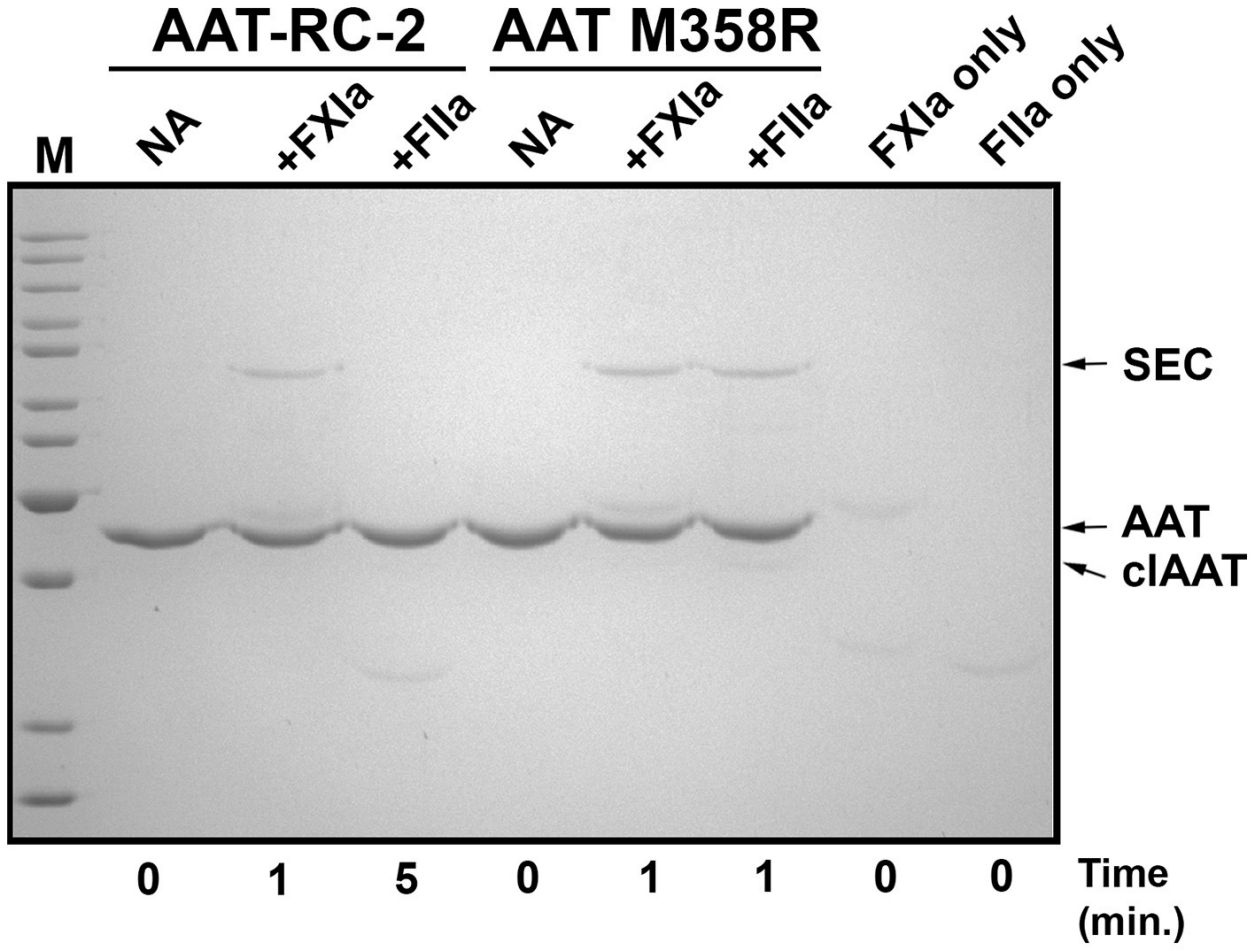
AAT-RC-2 forms sodium dodecyl sulfate (SDS)-stable complexes with FXIa but not with thrombin. A 10% SDS-polyacrylamide gel electrophoresed under denaturing and reducing conditions and stained with Coomassie Blue is shown. Reactions contained purified AAT-RC-2 or AAT M358R (identified above horizontal bars indicating the relevant lanes) in the absence (no addition, NA) or presence of FXIa (+FXIa) or thrombin (+FIIa). AAT variants (1.0 μM) were reacted with protease (0.1 μM) for the time, in minutes, indicated below the lanes, at 37°C. Arrows, at the right, highlight the position of intact AAT (AAT), cleaved AAT (clAAT), and serpin–enzyme complexes (SEC) of AAT-FXIa or AAT-thrombin. M denotes molecular weight markers, in kDa: 200, 150, 120, 100, 85, 70, 60, 50, 40, 30, and 25. The scanned image of the gel was uniformly darkened in Adobe Photoshop by setting the brightness to −50 (full scale 0 to −150) to maximize band visibility.

We next used gel-based assays to gain additional insights into the reaction of AAT-RC-2 with FXIa. We first examined the reaction products under pseudo-first-order conditions of 10-fold molar excess serpin over protease. As shown in Figure 2, and as anticipated based on the *k*_2_ observations in Tables 2, 3, AAT RC-2 and AAT M358R formed an SDS-stable complex with the light chain of FXIa, as shown by the appearance of an ~78-kDa protein band following incubation of excess AAT under conditions for 1 min [Figure 2, compare no addition (“NA”) lanes to “+FXIa” and “FXIa only” lanes]. Similarly, an SDS-stable complex was formed on incubation of AAT M358R with thrombin; no such complex formation was observed when AAT-RC-2 was incubated with thrombin, despite the lengthening of the reaction time to 5 min.

**Figure 3:**
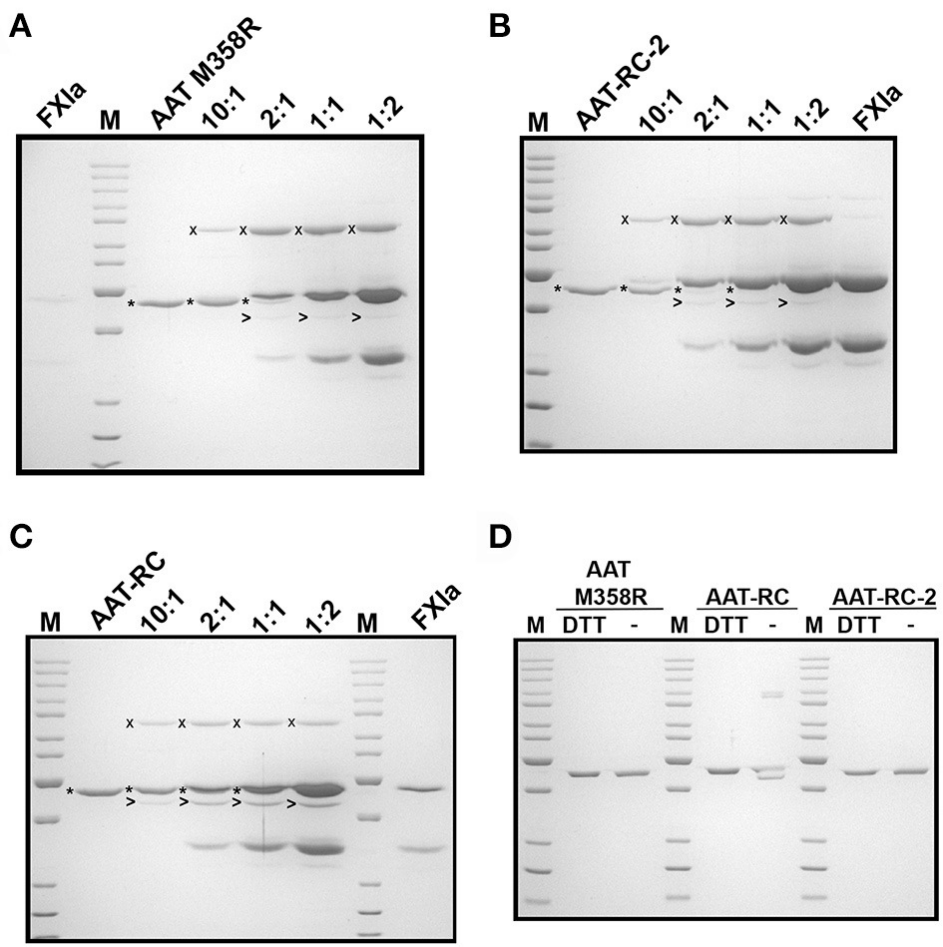
Effects of increasing amounts of FXIa on reaction outcomes with AAT variants and electrophoretic profiles. A 10% SDS-polyacrylamide gel electrophoresed under denaturing and reducing **(A–C)** or reducing and non-reducing conditions **(D)** and stained with Coomassie Blue is shown. AAT variants (1.0 μM) were incubated with or without 0.1–2.0 μM FXIa, yielding molar reaction ratios of AAT/FXIa shown above the lanes. The position of AAT-FXIa serpin–enzyme complexes (x), unreacted AAT (*), and cleaved AAT (>) is indicated to the left of the respective bands, where reactions with AAT M358R, AAT-RC-2, and AAT-RC are shown in **(A–C)**, respectively. FXIa-only lanes in **(A–C)** correspond to 0.1, 2.0, and 0.5 μM concentrations, respectively. **(D)** AAT variants identified above the lanes (0.7 μg per lane) were electrophoresed under reducing conditions (with dithiothreitol, DTT) or non-reducing conditions (–). M, molecular weight markers, are the same as those in Figure 1.

**Figure 4:**
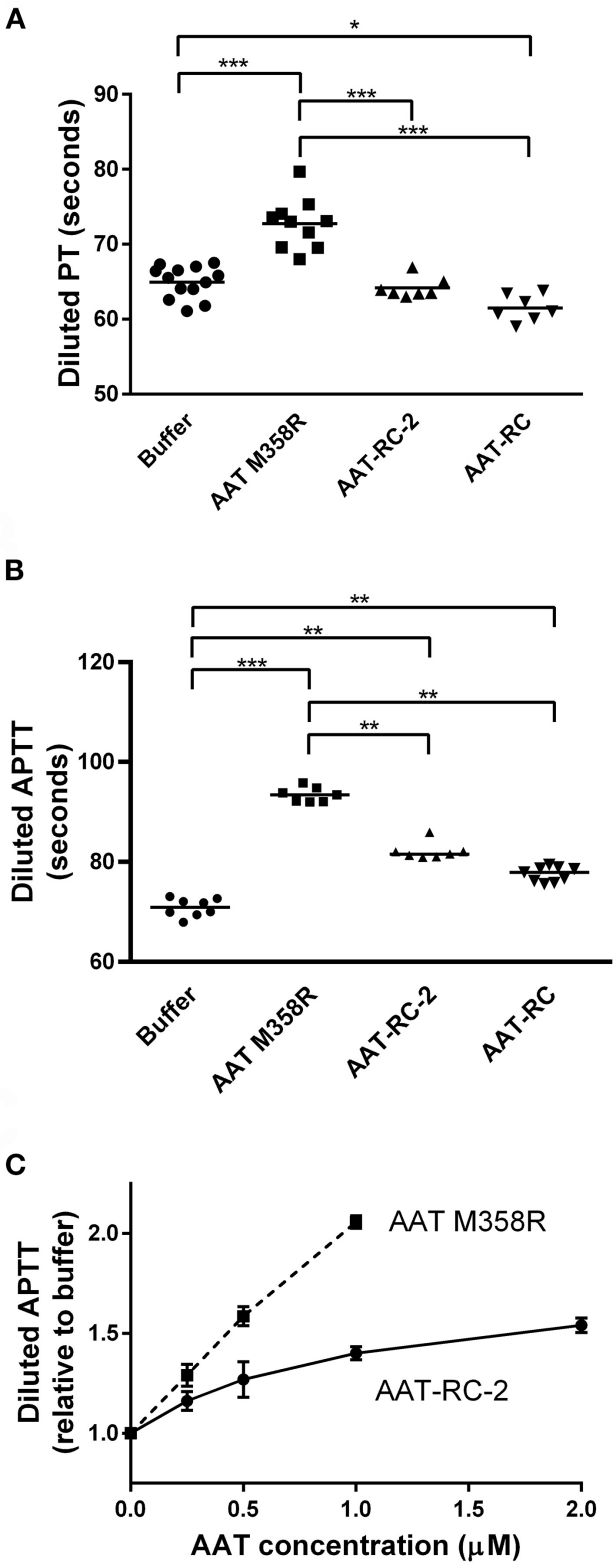
Effects of AAT variants on modified hemostasis screening tests. Buffer or purified AAT M358R was introduced at 0.5 μM final concentrations into diluted PT **(A)** or diluted APTT **(B)** assays. Note that the y-axis does not commence at 0 s on any graph. Each point represents a single determination. Horizontal capped lines that link different groups indicate statistical differences by ANOVA with post-tests: *p < 0.05; **p < 0.01; and ***p < 0.001. **(C)** A dose-response curve that shows diluted APTT values (normalized to buffer-only controls) as a function of increasing variant AAT concentration (μM). The mean ± SD of three determinations for each point is shown: squares, AAT M358R; circles, AAT-RC-2.

**Figure 5:**
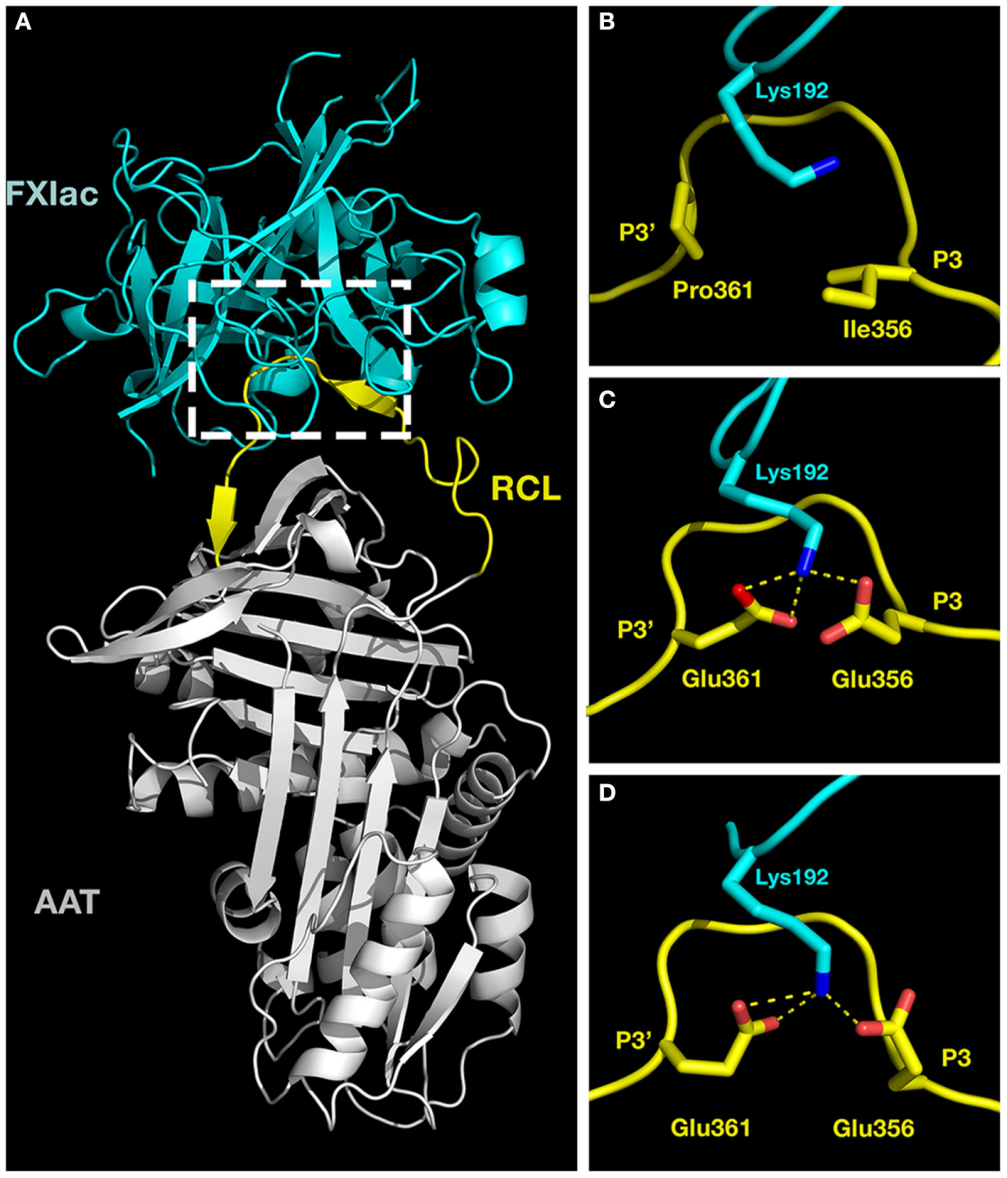
Molecular models of activated factor XI docked to AAT variants. Crystal structures of the catalytic domain of FXIa (FXIac, cyan, PDB 1ZNT) and AAT M358R (PDB 1OPH, RCL yellow, rest of AAT gray) were docked using ClusPro 2.0 as described in the *METHODS* section. **(A)** The full structure of the docked proteins. **(B–D)** Exploded close-ups of the AAT RCL and the active site of FXIac [corresponding to the white dashed box in **(A)**] for AAT M358R **(B)**, AAT-RC **(C)**, and AAT-RC-2 **(D)**. Yellow dashed lines indicate hydrogen bonds predicted to form between FXIac K192 nitrogen atoms (blue) and Glu 356 and Glu 361 oxygen atoms (red) in AAT-RC and AAT-RC-2 **(B,C)** but not in AAT M358R **(A)**. More remote portions of the FXIac chain were deliberately reduced in visibility using PyMOL in order to maximize the visibility of K192 of FXIac and the AAT RCL between P3 and P3' in **(B–D)**.

While the reactions shown in Figure 2 provided information about initial reaction products, they were uninformative concerning whether the different AAT variants were fully reactive with FXIa because of consumption of all the FXIa in the reaction. We therefore examined the reaction products at a range of FXIa concentrations, including molar excesses. As shown in Figure 3, decreasing the ratio of AAT/FXIa from 10:1 (same conditions as Figure 2) to 5:1 to 1:1 resulted in complete reaction of AAT M358R (Figure 3A) and AAT-RC-2 (Figure 3B); in the case of AAT-RC, the unreacted protein and the heavy chain of FXIa co-migrated, preventing visualization (Figure 3C). For all three AAT variants, formation of SDS-stable 78-kDa complexes was readily visualized, as was AAT cleavage to a protein species of ~45 kDa. Complex formation increased up to 1:1 ratios of AAT M358R or AAT-RC-2, although complex bands were less intense for AAT-RC. Conversely, cleaved AAT species were detectable at lower levels of FXIa for AAT-RC than the other two proteins, and the amount of cleaved product was noticeably greater at all ratios. Although the conditions used in the kinetic determination of SI and in these gel-based experiments were not identical, the results were qualitatively consistent in that the anti-FXI reactivity of AAT M358R and AAT-RC-2 was greater than that of AAT-RC, and the substrate behavior of AAT-RC (leading to cleaved AAT) exceeded that of these other two AAT variants.

AAT-RC exhibited a lower *k*_2_ value than AAT M358R or AAT-RC-2 and formed less complex under conditions in which more FXIa was added. As shown in Figure 3D, when AAT-RC was electrophoresed under non-reducing conditions, only a portion of the preparation co-migrated with its reduced counterpart. Other fractions of the preparation exhibited retarded migration (i.e., doublet band migrating between 85- and 100-kDa markers) consistent with intermolecular disulfide bond formation and accelerated migration consistent with intramolecular disulfide bond formation (i.e., band migrating more rapidly than that co-migrating under reduced and non-reduced conditions). These properties of AAT-RC were dependent on the presence of the F352C mutation, since both AAT M358R and AAT-RC-2 preparations were comprised of single bands that co-migrated irrespective of whether reducing agents were added (Figure 3D). Linkage of two RCLs by a disulfide bond would be expected to prevent inhibition of target proteases.

### AAT-RC-2 Prolongs Modified APTT but Not PT Clotting Times in Human Plasma

Having demonstrated using kinetic and gel-based assays that AAT-RC-2 maintains or exceeds the rapid rate of FXIa inhibition of AAT M358R, with the same reaction stoichiometry but greatly enhanced selectivity, we addressed the effects of AAT-RC-2 on the clotting of human plasma *in vitro*. We employed modified forms of the hemostasis screening assays (PT and APTT). Figure 4A shows that supplementing pooled human plasma with buffer or 0.5 μM AAT-RC or AAT-RC-2 failed to increase the clotting time provoked by recalcification and provision of diluted tissue factor-containing PT reagent. In contrast, supplementation with 0.5 μM AAT M358R significantly prolonged this clotting time from a baseline of 65 ± 2 s to 73 ± 3 s (mean ± SD, *p* < 0.001).

In an APTT assay modified by dilution of the silica activator reagent, supplementation with 0.5 μM AAT M358R, AAT-RC-2, or AAT-RC significantly prolonged the clotting time compared to buffer alone (Figure 4B). The prolongation elicited by AAT-RC-2 or AAT-RC did not differ but was less than that elicited by AAT M358R. While the experiments shown in Figures 4A,B were performed at a single 0.5-μM AAT dose, in Figure 4C, we demonstrated a dose response for both AAT M358R and AAT-RC-2 in the diluted APTT assay. The dose response of AAT-RC-2 was shallower than that of AAT M358R, as expected based on the greater effect of AAT M358R than AAT-RC-2 in the assays shown in Figure 2B.

### Molecular Modeling Suggests Hydrogen Bonding Between P3 and P3' Glutamates and K192 in FXIac for AAT-RC-2 and AAT-RC but Not AAT M358R

We sought molecular explanations for the selectivity of AAT-RC-2 and AAT-RC as FXIa inhibitors. We used the ClusPro 2.0 web server, which carries out iterative rigid body docking simulations coupled to energy minimizations to construct a model of such a complex (35, 36). Essentially, the atomic coordinates of FXIac and AAT M358R were extracted from their crystal structures in complex with the KPI-PN2 domain (PDB 1ZJD) (38) or trypsin S195A (PDB 1OPH) (26), respectively, and input as receptor and ligand for *in silico* docking. Figure 5A shows a model of the initial, non-covalent encounter (also called Michaelis) complex of the light chain catalytic domain of FXIa (FXIac, cyan) docked to AAT M358R (RCL, yellow, and rest of the serpin, gray). Inspection of this structural model revealed close contacts between the active site of FXIac and portions of the AAT M358R RCL, as expected. Following *in silico* mutation of the RCL to create models of AAT-RC and AAT-RC-2 and docking to FXIac, these areas (see dashed box, Figure 5A) were examined in greater detail. As shown in close-up Figure 5B, the side chains of I356 (P3) and P361 (P3') in AAT M358R and K192 of FXIac possess no significant electrostatic or hydrophobic interactions that would contribute to binding the RCL in the FXIa active site. In contrast, in AAT-RC (Figure 5C) or AAT-RC-2 (Figure 5D), mutation of I356 (P3) and P351 (P3') to glutamate in both AAT-RC and AAT-RC-2 predicts similarly docked models. Both models suggest the I356E and P361E mutations create a hydrogen bonding network that would stabilize the interaction between the FXIa active site and AAT-RC or AAT-RC-2.

We followed an analogous approach to that described above to focus on AAT residues 354–358 in AAT M358R, AAT-RC, and AAT-RC-2, using the covalent complex crystal structure of AAT and trypsin (PDB 1EXZ) and focusing on the AAT moiety alone (25). However, in contrast to the results in the encounter complex with K192, no differences between these structural models were found (data not shown).

## Discussion

The starting point for this investigation was our discovery of AAT-RC, a variant of AAT M358R containing five additional substitutions (F352C/A355V/I356E/I360T/P361E) at the P7, P4, P3, P2', and P3' positions of the serpin RCL (31). The variant residues between P7 and P3 inclusive were selected by biopanning an AAT M358R phage display library hypervariable at that portion of the RCL, while the rest of the AAT-RC additional substitutions were selected by lysate screening of a bacterial expression library of AAT M358R hypervariable at P2' and P3'. The two motifs were then combined in AAT-RC. In this investigation, we reversed the additional changes, one at a time, back toward the “parental” AAT M358R molecule. Our objective was to determine if AAT-RC was optimally selective for FXIa, and if one cost of its increased specificity, a 2.3-fold decrease in the rate of FXIa inhibition vs. AAT M358R, was unavoidable. If so, then each stepped reversal of mutation might have been expected to decrease FXIa selectivity and increase FXIa activity. Our experimental results, however, did not follow this linear path.

Reverting the P7 mutation F352C to the wild-type Phe residue led to a 13% decrease in selectivity for FXIa over kallikrein in AAT-RC-1, followed by a 31% increase in selectivity when the P4 mutation A355V was also reverted in AAT-RC-2 and a substantial 711% drop in selectivity when I356E and I360T were reversed in AAT-RC-3. Although in this study we did not examine AAT-RD, intermediate in mutation reversal between AAT-RC-2 and AAT-RC-3, its 7-fold decrease in the rate of FXIa inhibition (31), to the lowest *k*_2_ value of any of the variants in the mutational series depicted in Table 1, reinforces the non-additive nature of RCL mutations in AAT M358R. Such cooperativity was previously noted by Hopkins et al. who substituted the RCL of antithrombin for that of AAT M358R in AAT and then back-mutated this variant to generate an array of less modified AAT constructs (40). These investigators noted that changes in different parts of the RCL had greater effects when combined than when separate, with respect to APC inhibition, just as we observed with FXIa inhibition.

Our approach in this study and its predecessor (31) relied heavily on the determination of *k*_2_ values and the reporting of selectivity as the ratio of the rate constants for the inhibition of two different proteases by a particular AAT variant. It should be noted that *k*_2_ is an aggregate term reflecting the association rate constant diminished proportionately by non-productive turnover (e.g., substrate behavior leading to AAT cleavage and protease escape). We assessed non-productive turnover by measuring the SI. SI elevations could have resulted from a subset of inhibitor molecules being unable to react with the inhibitor, impairments in the inhibitory mechanism leading to serpin cleavage, or instability of the serpin–enzyme complex. Gel-based analyses using a range of AAT/FXIa ratios showed that both AAT M358R and AAT-RC-2 reacted fully with FXIa, proceeding down the branched pathway to either complexed or cleaved serpin outcomes. In contrast, AAT-RC did not appear to react fully and was non-productively cleaved to a greater extent than the other two AAT variants. These relative deficiencies of AAT-RC likely arose from the formation of intermolecular disulfide bonds linking two AAT-RC molecules via the F352C cysteine residue in their RCLs. Such RCL-linked dimers, whose formation we demonstrated via non-reducing electrophoresis, would be expected to be unable to form serpin–enzyme complexes. Two dimeric forms of AAT-RC were detected via non-reducing electrophoresis; these likely represented intermolecular disulfide bonding between C352 and C352 and between C232 and C352, since wild-type AAT contains a single Cys residue at position C232 (41). Intramolecular disulfide bond formation was also suggested by the presence of a more rapidly migrating AAT-RC species not observed under reducing conditions, likely intramolecular C232–C352. Such a misfolded species would also be highly unlikely to form productive complexes with proteases; a precedent exists in antitrypsin I (R39C) (42). Taken together, these intermolecular and intramolecular oxidized forms of AAT-RC likely account for most of the difference in SI between AAT-RC and AAT-RC-2 or AAT M358R. Those AAT-RC molecules not diverted into non-productive dimers might also been converted into cleaved form to a greater extent than AAT M358R or AAT-RC-2 because F352C would disrupt an interface formed by F352 and a patch of hydrophobic residues underlying helix F observed in crystal structures of RCL-inserted AAT, either free or complexed to proteases (25, 26, 43).

AAT-RC-2 differs from AAT M358R at three positions: I356E (P3), I360T (P2'), and P361E (P3'). While inspection of the aligned RCL sequences of 30 human serpins shows Thr at P2' in two serpins (SERPIN A12, vaspin, and SERPIN D1, heparin cofactor II), Glu is not found at P3 or P3' in any of these serpins (44). Given the more conservative nature of the I360T substitution, our previous finding that an I360T/P361Q substitution enhanced thrombin reactivity of AAT M358R (45), and the diametrically opposed decreases in thrombin reactivity of AAT-RC and AAT-RC-2 and increases in FXIa reactivity of these proteins, it is likely that I356E and P361E (P3') are primarily responsible for the desirable properties of AAT-RC-2 as a selective FXIa inhibitor. While there is some precedent for FXIa favoring Glu at P3, in that FXIa cleaves the bond following EPR in an amyloid beta protein precursor (46), in engineered activatable hirudin-based thrombin inhibitors (47, 48), and in a chloromethyl ketone chromogenic substrate with specificity for FXIa (49), no precedent exists for a favorable interaction with Glu at P3'.

To gain greater understanding into the interactions of AAT-RC-2 with FXIa, we turned to *in silico* protein structural modeling. No crystal structure of any form of AAT in complex with FXIa can be found in the Protein Data Bank. However, AAT has been crystallized in two complexed forms: in M358R form, as an encounter complex with active site-mutated S195A trypsin (24), and in wild-type form, as a cleaved, covalently bonded serpin-enzyme complex with trypsin (25) or porcine pancreatic elastase (26). We chose to model an initial encounter complex between inhibitor and protease to understand how the changes we had engineered improved AAT M358R reactivity with and specificity for FXIa. The catalytic domain of FXIa has been crystallized with a number of inhibitors; we chose the complex of FXIac with the small protein Kunitz protease inhibitor domain of protease nexin 2 (38). We extracted the AAT M358R structure (24) and the FXIac structure (38) from these complexes and docked them using ClusPro 2.0 (35–37). We also introduced the mutations from AAT-RC and AAT-RC-2 *in silico*, minimizing steric clashes using PyMOL, docked them, and compared the three modeled complexes. The main difference that we noted was with respect to interaction of the variant AAT protein RCLs with K192, a residue N-terminally adjacent to the conserved GD*S*GGP motif surrounding the active site serine (S195 in chymotrypsin convention numbering, underlined) in serine proteases (50). We noted the stabilization of the interaction between E356 and E361 in AAT-RC and AAT-RC-2 by three hydrogen bonds not capable of forming in AAT M358R between K192 and the native P3 I356 and P361 residues. Notwithstanding slight differences in predicted hydrogen bond lengths and angles between FXIa K192 and AAT E356 and E361 in AAT-RC and AAT-RC-2, these models suggest that the encounter complex between AAT-RC and FXIa would have been favored to a similar extent as that between AAT-RC-2 and FXIa, had the former been able to form without interference from disulfide bonded inter-RCL dimerization.

It should be noted that ClusPro is limited in its predictive ability compared to molecular dynamics with free energy perturbation calculations. While molecular dynamics would provide a higher quality model, such approaches are beyond the scope of this study. We improved the docking model outcome from ClusPro by partially defining the interacting interface (AAT RCL and residues of FXIac) based on experimental evidence of the FXIac interacting interface from the co-crystal structure of FXIac with KPI-PN2 and mutagenesis studies (38, 39). Regardless, the proposed interactions of AATA-RC-2 E356 and E361 with FXIa K192 should be regarded as a hypothesis suggested by one modeling approach, one that could be tested in future via either molecular dynamics or experiments with recombinant FXI molecules altered at K192. Inspection of 79 proteins representing the S1A trypsin subfamily of human serine proteases revealed that Lys residues were uncommon at position 192 in coagulation-related proteases (51). Q192 is found in FXIIa and FXa and E192 in APC and thrombin. The latter residues might be expected to repel E356 and E361 in AAT-RC and AAT-RC-2. Of the proteases tested in this study, only kallikrein contains a Lys residue at position 192. However, we observed that AAT-RC-2 had a greatly elevated SI for kallikrein compared to that of AAT M358R. Thus, had the encounter complex formed efficiently between AAT-RC-2 and kallikrein, it is highly unlikely that it would have proceeded to form a stable covalent complex. Other spatial conflicts must have prevented the rapid insertion of the AAT-RC-2 RCL, but not the AAT M358R RCL into underlying β-sheet A, since failure to retain captured protease in a stable complex correlates with a slowed speed of cleaved RCL (N-terminal to P1) insertion (30).

AAT-RC-2 is unusual among recently engineered AAT M358R variants in that it appears to exhibit enhanced selectivity for its target protease, without the cost of decreased activity. Polderdijk et al. reported a 7-fold decrease in the rate of APC inhibition for KRK AAT, an AAT M358R variant engineered to inhibit APC selectively, which was unreactive with thrombin and which inhibited FXIa and FXa with rate constants reduced by factors of 850 and 360, respectively (52). De Maat et al. exchanged tripeptide SMT for AIP in the P4-P2 positions of AAT M358R as well as S359V in AAT SMTRV to generate a variant AAT engineered to inhibit contact pathway proteases. While this variant inhibited kallikrein 2.5-fold more rapidly than AAT M358R, and was 20-fold less effective at inhibiting plasmin, these enhancements came at the cost of a 1.8-fold reduction in its rates of FXIa inhibition (34).

The effects of AAT-RC-2 in human plasma correlated with its retained AAT M358R-like rate of FXIa inhibition and its loss of AAT M358R's ability to inhibit other coagulation proteases. Neither AAT-RC nor AAT-RC-2 prolonged the diluted PT; the prolongation by AAT M358R likely arose due to its anti-FXa and anti-thrombin capacities, since the PT is affected by the extrinsic and common pathways of coagulation (53). Some prolongation of the diluted APTT by AAT-RC-2 and AAT-RC was observed, consistent with their anti-FXIa capacities, but it again did not surpass that of AAT M358R because of that inhibitor's effects on both contact factor and common pathways (53). The greater anticoagulant activity of AAT-RC-2 than AAT-RC in the FXI-dependent APTT likely reflected the more rapid inhibition of FXIa by AAT-RC-2 than AAT-RC in this more sensitive assay, as well as the diminished reactivity of AAT-RC arising from intermolecular or intramolecular disulfide bond formation. de Maat et al. reported a similar intermediate effect of AAT variant SMTRV on the diluted APTT, greater than buffer and lesser than AAT M358R, consistent with its anti-FXIa and anti-FXIIa activities (34).

AAT-RC-2 marks the culmination of our mutagenic campaign to engineer AAT M358R into a specific FXIa inhibitor. This goal was achieved with the alteration of only three additional residues. We employed phage display and bacterial lysate screening to probe three sectors of the RCL and then combinatorial mutagenesis to arrive at AAT-RC (31) and back-mutation to define the minimum mutations necessary to maintain FXIa specificity. While complex, this strategy permitted the engineering of the inhibitor without *a priori* assumptions and was vindicated by the cooperativity observed between changes in different residues in the AAT M358R RCL. AAT-RC-2 should function as a specific FXIa inhibitor *in vivo* when it is tested in animal models. The effectiveness of KRK AAT and SMTRV AAT *in vivo*, specifically in countering the hemorrhagic tendency of FIX knockout mice for KRK AAT (52) and in reducing the thrombotic and inflammatory responses of normal mice treated with SMTRV AAT (34), may bode well for future *in vivo* experimentation with AAT-RC-2.

## Data Availability Statement

The original contributions presented in the study are included in the article/Supplementary Material, further inquiries can be directed to the corresponding author/s.

## Author Contributions

WS conceived and designed the study, secured competitive funding to support it, and wrote the manuscript. MH contributed to writing the manuscript. MH and VB performed the experiments. MH and WS created the figures. MH conducted all protein modeling studies under the direction of SA. All authors contributed to the data analysis, involved in the editing and revision of the manuscript, and approved its final version.

## Conflict of Interest

The authors declare that the research was conducted in the absence of any commercial or financial relationships that could be construed as a potential conflict of interest.
